# Structure Properties of Generalized Farey graphs based on Dynamical Systems for Networks

**DOI:** 10.1038/s41598-018-30712-2

**Published:** 2018-08-15

**Authors:** Wenchao Jiang, Yinhu Zhai, Paul Martin, Zhiming Zhao

**Affiliations:** 1 School of Computer, Guangdong University of Technology, Guangzhou, 510006 China; 2 School of Information Engineering, Guangdong University of Technology, Guangzhou, 510006 China; 3 System and Network Engineering research group, Informatics Institute, University of Amsterdam, Science Park 904, 1098XH Amsterdam, The Netherlands

## Abstract

Farey graphs are simultaneously small-world, uniquely Hamiltonian, minimally 3-colorable, maximally outerplanar and perfect. Farey graphs are therefore famous in deterministic models for complex networks. By lacking of the most important characteristics of scale-free, Farey graphs are not a good model for networks associated with some empirical complex systems. We discuss here a category of graphs which are extension of the well-known Farey graphs. These new models are named generalized Farey graphs here. We focus on the analysis of the topological characteristics of the new models and deduce the complicated and graceful analytical results from the growth mechanism used in generalized Farey graphs. The conclusions show that the new models not only possess the properties of being small-world and highly clustered, but also possess the quality of being scale-free. We also find that it is precisely because of the exponential increase of nodes’ degrees in generalized Farey graphs as they grow that caused the new networks to have scale-free characteristics. In contrast, the linear incrementation of nodes’ degrees in Farey graphs can only cause an exponential degree distribution.

## Introduction

There are two types of common feature that exist in real-life complex networks: one is the scale-free distribution of degree, and the other is small-world behavior. To mimic the two main features existing in most real-life networks, researchers have proposed a wide variety of models. Random networks models, including the famous WS small-world models^1^ and BA scale-free networks^2^, stimulated an in-depth understanding of the various physical mechanisms in empirical complex networks from 1998 onwards. However, the uncertain creation mechanism and huge computation requirements for analysis are two main shortcomings of random models. Deterministic models can be designed that have the same important properties similar as random models, such as being scale-free, small-world and highly clustered, and thus can be used to imitate empirical networks appropriately. Hence the study of the deterministic models of complex networks has increased recently.

The first deterministic model is deduced by Comellas^3^; it presents the property of being small-world. Barabási confirms that the scale-free property can also emerge in deterministic networks, just as it emerges in random models^4^. Deterministic uniform recursive tree is a deterministic version of the random uniform recursive tree; Zhang studies the topological characteristics and spectral properties of the Laplacian matrix in it^5^. Moreover, inspired by the simple recursive operation, techniques of plane filling and generating processes of fractals, several deterministic models^6–14^ have been created and studied carefully.

Recently, the famous Farey sequence of irreducible fractions between 0 and 1 has been related to graph constructions known as Farey graphs^15^. The graphs have many interesting properties: they are minimally 3-colorable, uniquely Hamiltonian, maximally outerplanar and perfect^15^. Researchers show that Farey graphs are an appropriate model for networks associated with some complex systems^16–19^.

We here propose another family of complex network based on Farey graphs, named generalized Farey graphs. We rigorously derive the main topological properties of generalized Farey graphs found in such graphs, including average degree, degree distribution, clustering coefficient, network diameter and average path length. We find that our generalized Farey graphs are different from Farey graphs in that they possess the new property of being scale-free. In other words, the new models are more suitable for depicting and revealing complexity and universality of complex network systems than standard Farey graphs.

## Definition, Order and Size of Generalized Farey Graphs

To ensure the integrity of the paper, we here duplicate the construction method and main properties of Farey graphs found in ref.^15^. Farey graphs are constructed in an iterative manner; let ${F}_{t}$ be the Farey graph after $t\in N$ iterations.

### *Definition 1*.

Farey graph ${F}_{t}$, for $t\ge 0$, is constructed as follows:

For $t=0$, ${F}_{0}$ has two vertices and a single edge joining them together.

For $t\ge 1$, ${F}_{t}$ is obtained from ${F}_{t-1}$ by adding a new vertex adjacent to both the end vertices of every edge introduced at step $t-1$.

### *Remark 2*.

**Figure 1:**
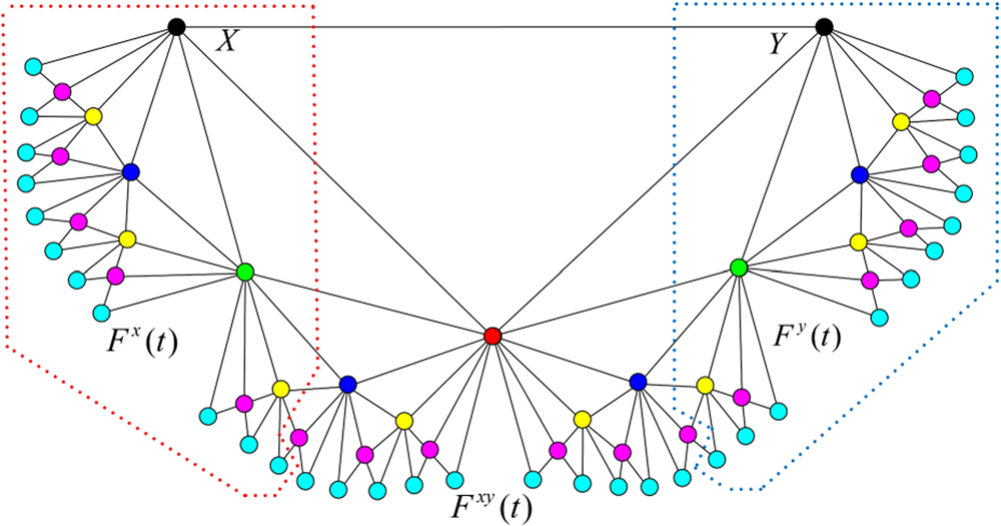
Farey graph ${F}_{6}$. The vertices, which are added to ${F}_{6}$ at time from $t=0$ to 6, are marked with different colors: black, red, green, blue, yellow, purple and cyan, respectively.

Figure 1 shows the case of a Farey graph after step $t=6$. The number of vertices and edges of the graph ${F}_{t}$ are ${2}^{t}+1$ and ${2}^{t+1}+1$ respectively. The average degree is $4-(3/(2t+1))$; for large *t*, it is small and approximately equal to 4. The cumulative degree distribution follows an exponential distribution ${P}_{cum}(k) \sim {2}^{-\frac{k}{2}}$. The clustering coefficient is $c(F(t))=\frac{1}{{2}^{t}+1}[{2}^{t}\,\mathrm{ln}\,2-\frac{1}{2}{\rm{\Phi }}(\frac{1}{2},1,1+t)+\frac{1}{t+1}]$, where ${\rm{\Phi }}$ denotes Lerch’s transcendent function; the clustering coefficient tends to $\mathrm{ln}\,2$ for large value of *t*, thus the clustering coefficient of ${F}_{t}$ is high. The diameter is $diam({F}_{t})=t$, $t\ge 1$, and the average path length is $\mu ({F}_{t})=\frac{1}{9({2}^{t}+1)}({2}^{t}(5+{(-1)}^{t}+(6t+17){2}^{t}+$ $(6t-5){4}^{t}))$; therefore the Farey graph is a small-world network.

### *Remark 3*.

The two hub vertices in ${F}_{t}$ are marked with *X* and *Y*. By then comparing the two distances from itself to the two hub nodes, all the nodes in ${F}_{t}$ can be divided into three parts: ${F}^{x}(t)$, ${F}^{y}(t)$ and ${F}^{xy}(t)$. Each node in ${F}^{x}(t)$ has shorter distance from itself to *X* than to *Y*. ${F}^{x}(t)$ in Fig. 1 is enclosed by the red dotted line. On the contrary, the nodes in ${F}^{y}(t)$ have shorter distance to *Y* than to *X*, which are enclosed by the blue dotted line in Fig. 1. The remaining nodes are assigned to ${F}^{xy}(t)$, in which the two distances are equal. From this point of view, $F(t)={F}^{x}(t)\cup {F}^{xy}(t)\cup {F}^{y}(t)$. It is worth pointing out that the number of nodes in ${F}^{x}(t)$ and ${F}^{y}(t)$ are equal for every Farey graph due to the symmetry of their construction.

Although several papers show that Farey graphs are a good model for networks associated with some complex systems^16–19^, but they have not the important characteristic of being scale-free. In this paper, we generalize the construction method of Farey graphs, to construct *generalized Farey graphs*, and we focus on analytic solutions to derive their topological properties. The construction of generalized Farey graphs is shown below.

### *Definition 4*.

The generalized Farey graph ${G}_{m,t}$ (where $m\in {\rm{N}}$) is constructed in an iterative way:

For $t=0$, ${G}_{m,0}$ is a triangle whose three vertices connect one another;

For $t\ge 1$, ${G}_{m,t}$ is obtained from ${G}_{m,t-1}$ by adding for each edge created at step $t-1$ an additional *m* new vertices, and attaching the *m* new nodes to both end vertices of that edge.

### *Remark 5*.

**Figure 2:**
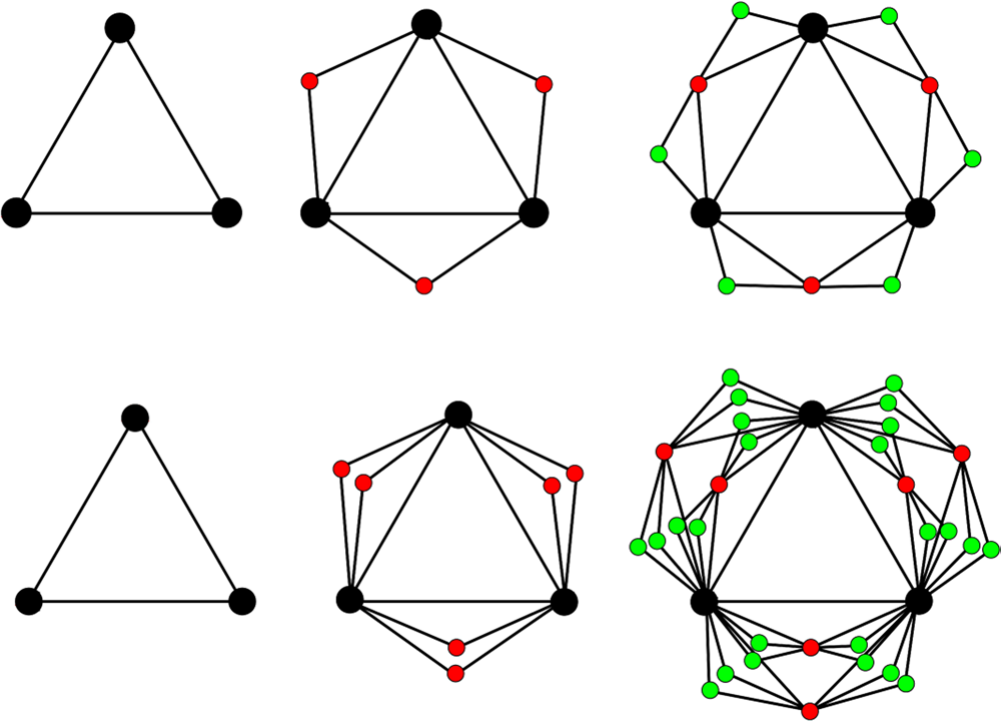
The first three steps of generalized Farey graphs ${G}_{m,t}$, when $m=1,2$ and $t=0$, 1, 2, respectively. (**a**) The generalized Farey graphs ${G}_{1,0}$, ${G}_{1,1}$ and ${G}_{1,2}$. (**b**) The generalized Farey graphs ${G}_{2,0}$, ${G}_{2,1}$ and ${G}_{2,2}$.

Figure 2 shows the first three steps of the construction of ${G}_{m,t}$ in the cases of $m=1$ and 2. If *m* = 1, ${G}_{m,t}$ degenerates into the union of three Farey graphs ${F}_{t}^{i}$ (*i = *1, 2 and 3), created by merging a different hub node from each Farey graph pair ${F}_{t}^{i}$ and ${F}_{t}^{j}$(*i* ≠ *j*) to create three shared hub nodes for the generalized Farey graph (see Fig. 2a).

**Figure 3:**
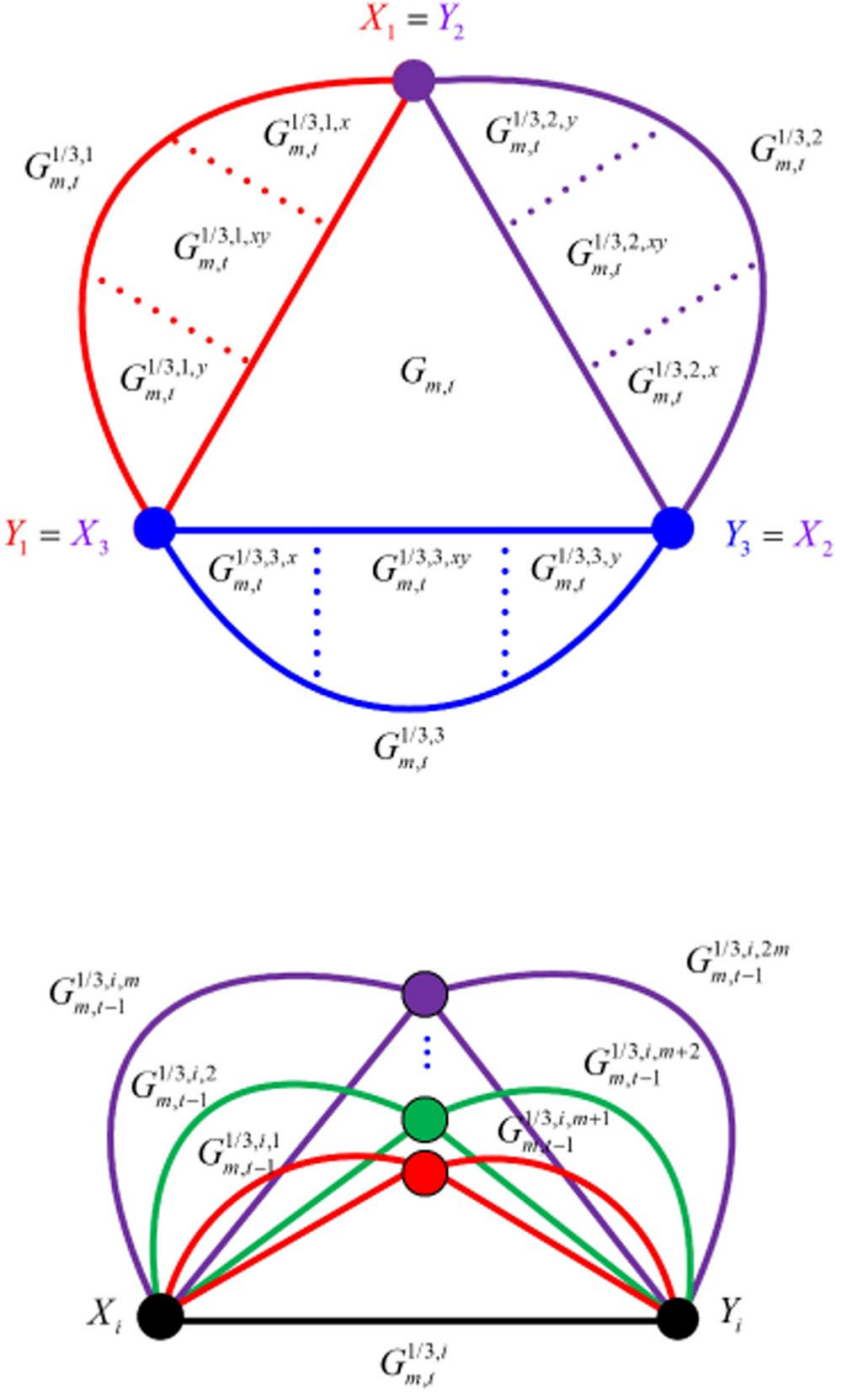
The detailed construction patterns in ${G}_{m,t}$ and ${G}_{m,t}^{1/3,i}$. (**a**) ${G}_{m,t}$ is the merging of three sub-networks: ${G}_{m,t}^{1/3,i}$, where $i=1,2,3$. (**b**) ${G}_{m,t}^{1/3,i}$ is constructed by *2 m* copies of ${G}_{m,t-1}^{1/3,i,j}$, where $i=1,2,3$ and $j=1,2,\mathrm{...},2m$.

Next, we study the detailed structure of ${G}_{m,t}$, which is shown in Fig. 3.

Firstly, as shown in Fig. 3a, ${G}_{m,t}$ is divided into three sub-networks, denoted as ${G}_{m,t}^{1/3,i}$, $i=1,2,3$. Every sub-network shares with each of the other sub-networks a common hub. As described in Remark 5, ${G}_{m,t}$ is exactly the combination of three equivalent sub-networks, created by merging six hubs into three nodes in a triangle arrangement. Just as for a Farey graph, each sub-network ${G}_{m,t}^{1/3,i}$ has two initial nodes, ${X}_{i}$ and ${Y}_{i}$, and all the nodes in it can be divided into three parts based on the distance from each node to the two hubs: ${G}_{m,t}^{1/3,i,x}$, ${G}_{m,t}^{1/3,i,xy}$ and ${G}_{m,t}^{1/3,i,y}$.

Secondly, based on the construction pattern used to create ${G}_{m,t}$, we can infer that ${G}_{m,t}^{1/3,i}$ is also recursively constructed by $2m$ copies of ${G}_{m,t-1}^{1/3,i,j}$, in which $j=1,2,\mathrm{...},2m$. This combination method is shown in Fig. 3b.

We now infer the number of vertices and edges in generalized Farey graphs ${G}_{m,t}$.

We first denote ${\delta }_{v}(t)$ and ${\delta }_{e}(t)$ as the number of new vertices and edges which are added to ${G}_{m,t}$ at step *t*. The number of vertices and edges in ${G}_{m,t}$ are therefore ${n}_{m,t}=\sum _{i=0}^{t}{\delta }_{v}(i)$ and ${e}_{m,t}=\sum _{i=0}^{t}{\delta }_{e}(i)$ respectively.

Using the construction method above, we deduce that, when $t > 0$:

1 $${\delta }_{v}(t)=\frac{1}{2}\times {\delta }_{e}(t),$$

2 $${\delta }_{e}(t)=2m\times {\delta }_{e}(t-1).$$

Combined with the initial conditions ${\delta }_{v}(0)=3$ and ${\delta }_{e}(0)=3$, we prove that, when $t > 0$:

3 $${\delta }_{v}(t)=\frac{3}{2}{(2m)}^{t},$$

4 $${\delta }_{e}(t)=3\times {(2m)}^{t}.$$

We can then infer that:

5 $${n}_{m,t}=\frac{3m\times {(2m)}^{t}+3m-3}{2m-1},$$

6 $${e}_{m,t}=\frac{6m\times {(2m)}^{t}-3}{2m-1}.$$

Thus, the average degree $< k{ > }_{m,t}=\frac{2{e}_{m,t}}{{n}_{m,t}}$ can be calculated as follows:

7 $$ < k{ > }_{m,t}=4-\frac{4m+6}{m\times {(2m)}^{t}+m-1}.$$

For large values of *t*, the average degree is also small and approximately equal to 4, same as regular Farey graphs. We can also see that the proposed new models are sparse network. The reason for this is that the largest proportion of nodes in two graphs ${F}_{t}$ and ${G}_{m,t}$ have only a degree of 2 (being newly added).

## Relevant Characteristics of Generalized Farey Graphs

In the following section we concentrate on the degree distribution, clustering coefficient, network diameter and average path length of generalized Farey graphs. Thanks to the deterministic nature of ${G}_{m,t}$, we can give exact values for the relevant topological properties of this graph family for different values of *t*.

### Degree distribution

The degree distribution is one of the most important statistical characteristics of a network. By the definition, the degree of a node *v* is the number of edges incident from *v*. We denote the degree of a node *v* originally added to graph ${G}_{m,t}$ at iteration ${t}_{i}\,(0\le {t}_{i}\le t)$ by ${k}_{v}({t}_{i})$. Referring to the creation mechanism for generalized Farey graphs defined earlier, we deduce that:

8 $${k}_{v}({t}_{i})=\frac{2({m}^{t-{t}_{i}}-1)}{m-1}.$$

Obviously, when *m* is larger than 1, any node’s degree increases exponentially. When *m = *1, the degree is ${k}_{v}({t}_{i})=2(t-{t}_{i}+1)$, which is a linear increase^15^.

Furthermore, the degree spectrum of the network is discrete. By simplifying ${k}_{v}({t}_{i})$ as *k*, and from equation (8), we obtain:

9 $${t}_{i}=t-\frac{1}{\mathrm{ln}\,m}\,\mathrm{ln}(\frac{m-1}{2}k+1).$$

The degree distribution $P(k)$ for a network gives the probability that a randomly selected vertex has exactly *k* edges. In the analysis of the degree distribution of real life networks, it is usual to consider their cumulative degree distribution, ${P}_{cum}(k)=\sum _{i=k}^{\infty }P(i)$. The probability that the degree of a vertex is greater than or equal to *k* corresponds to the cumulative degree distribution. The earlier that the vertices are added to the network, the higher the degree they will have. Hence, the cumulative degree distribution is the sum of all vertices added to the graph from steps $t=0$ to ${t}_{i}$, such that ${P}_{cum}(k)=\frac{1}{{n}_{m,t}}\sum _{i\le {t}_{i}}{\delta }_{v}(i)$. It follows that:

10 $${P}_{cum}(k)=\frac{3+3m+\mathrm{...}+\frac{3}{2}{(2m)}^{{t}_{i}}}{\frac{3m\times {(2m)}^{t}+3m-3}{2m-1}}=\frac{{(2m)}^{{t}_{i}}+m-1}{{(2m)}^{t}+m-1}.$$

Substituting equation (9) for ${t}_{i}$ in this expression gives:

11 $${P}_{cum}(k)=\frac{{(2m)}^{t}{(2m)}^{-\frac{\mathrm{ln}(\frac{m-1}{2}k+1)}{\mathrm{ln}m}}+m-1}{{(2m)}^{t}+m-1}.$$

Applying the rule ${a}^{-\frac{\mathrm{ln}b}{\mathrm{ln}c}}={b}^{-\frac{\mathrm{ln}a}{\mathrm{ln}c}}$, we can infer than that

12 $$\begin{array}{c}{P}_{cum}(k)=\frac{{(2m)}^{t}{(\frac{m-1}{2}k+1)}^{-\frac{\mathrm{ln}2m}{\mathrm{ln}m}}+m-1}{{(2m)}^{t}+m-1}\\ \,\,\,\approx \,{(\frac{m-1}{2})}^{-\frac{\mathrm{ln}2m}{\mathrm{ln}m}}{k}^{-\frac{\mathrm{ln}2m}{\mathrm{ln}m}},\,{\rm{for}}\,{\rm{large}}\,{t}.\end{array}$$

From the principle in ref.^2^, when considering the relation between the cumulative degree distribution and the degree distribution as a histogram of the probability density, the size of histogram bins (i.e., the separation between adjacent *k* values) is proportional to *k* itself. Therefore, for the cumulative degree distribution, ${P}_{cum}(k) \sim {k}^{-\gamma +1}$ means the degree distribution follows a power-law form $P(k) \sim {k}^{-\gamma }$^2^. For equation 12, we obtain that $P(k) \sim {k}^{-\gamma }$ with the exponent $\gamma =1+\frac{\mathrm{ln}\,2m}{\mathrm{ln}\,m}$, which belongs to the interval [2, 3]. When *m* increases from 2 to infinity, *γ* decreases from 3 to 2. It should be stressed that the exponent of degree distribution of most real scale-free networks also lies in the same range between 2 and 3^2,3^.

The scale-free characteristic emerges from generalized Farey graphs when *m* is larger than 1, and this property is not found in Farey graphs in which *m* = 1. Clearly, the scale-free property originates from the exponential increase of nodes’ degrees in generalized Farey graphs, instead of the linear incrementation found in regular Farey graphs.

### Clustering coefficient

The clustering coefficient defines a measure of the level of cohesiveness around any given node. Supposing any node *v* has ${k}_{v}$ neighbor nodes, and there are ${{\rm{\Delta }}}_{v}$ edges among these neighbors; the maximum possible value of ${{\rm{\Delta }}}_{v}$ is then ${k}_{v}({k}_{v}-1)/2$. The clustering coefficient ${c}_{v}$ of node *v* is therefore twice the ratio between them:

13 $${c}_{v}=\frac{2{{\rm{\Delta }}}_{v}}{{k}_{v}({k}_{v}-1)}.$$

The clustering coefficient of the whole graph is the average of all clustering coefficients individual nodes. Therefore, we next compute the clustering coefficient of every node in a generalized Farey graph and their average value.

Suppose that a node *v* is added to the graph at iteration ${t}_{i}$. From the definition of clustering coefficient, ${{\rm{\Delta }}}_{v}$ can also denote the number of triangles (connected triplets of nodes) added to the graph at iteration ${t}_{i}$ by the addition of this node. We then get the relationship:

14 $${{\rm{\Delta }}}_{v}=1+2m+\mathrm{...}+2{m}^{t-{t}_{i}}=\frac{2{m}^{t-{t}_{i}+1}-m-1}{m-1}.$$

Using the abbreviating ${k}_{v}={k}_{v}({t}_{i})$ and substituting equation (8) into (13), we obtain:

15 $${c}_{v}=\frac{\frac{4{m}^{t-{t}_{i}+1}-2m-2}{m-1}}{{k}_{v}({k}_{v}-1)}=\frac{m-1}{{m}^{t-{t}_{i}+1}-1}=\frac{2}{{k}_{v}}.$$

This expression indicates that the local clustering scales is ${c}_{v} \sim {{k}_{v}}^{-1}$.

The clustering coefficient (${C}_{t}$) of the whole network at arbitrary step *t* can then be easily computed:

16 $${C}_{t}=\frac{\sum _{v=0}^{t}{n}_{v}\times {c}_{v}}{{n}_{t}}=\frac{3(m-1)(2m-1)}{2m}\times \frac{\sum _{{t}_{i}=0}^{t}\frac{{(2m)}^{{t}_{i}}}{{m}^{t-{t}_{i}}-\frac{1}{m}}}{3m\times {(2m)}^{t}+3m-3}.$$

For large values of *m*, we get:

17 $${C}_{t}\approx \frac{(m-1)(2m-1)}{2{m}^{2}(2{m}^{2}-1)}\times \frac{{(2{m}^{2})}^{t+1}-1}{{(2{m}^{2})}^{t}+{m}^{t}}.$$

The clustering coefficient ${C}_{t}$ tends to $1-\frac{3m+2}{2{m}^{2}-1}$ for large value of *t*. Therefore, for large value of *t* and *m*, the clustering coefficient approaches a constant value 1. We know that only the complete graph has a clustering coefficient of 1. In contrast, the value for regular Farey graphs is $\mathrm{ln}\,2$^15^. Node degrees increase exponentially as generalized Farey graphs grow, while the growth for regular Farey graphs is linear, which leads to generalized Farey graphs having higher clustering coefficients than Farey graphs. The basic motif in each growth step is the creation of new closed triplets of nodes, which causes high clustering coefficients both in generalized Farey graphs and Farey graphs.

### Diameter

The longest shortest path between all pairs of nodes is called the diameter. Diameter is one of the most important evaluation indexes because it characterizes the maximum communication delay in the network. Below we give the precise analytical computation of the diameter of ${G}_{m,t}$ denoted by $Diam({G}_{m,t})$.

From Proposition 3.7 in ref.^15^, the diameter of a Farey graph ${F}_{t}$ is $Diam({F}_{t})=t$ when $t\ge 1$. That is to say, the distance from any vertex to an initial vertex, which is added to graphs at time $t=0$, is less than or equal to *t*.

By examining the structured construction of ${G}_{m,t}$ in Fig. 3a, if the shortest distance between any two vertices goes through an initial vertex, the distance between any pair of vertices is less than or equal to 2*t* + 1,

18 $$Diam({G}_{m,t})=2t+1.$$

Because the network diameter is proportional to the logarithm of the network nodes, just liking Farey graphs, generalized Farey graphs are small world networks, as well.

### Average path length

One of the most important properties of complex networks is average path length. Average path length is the average value of the distances (the shortest path length) between every two nodes in the network, and it is much more difficult to calculate than connectivity. Most real networks have been shown to be small-world or ultra-small-world networks, that is, their average path length behaves as a logarithmic or double logarithmic scaling with the network size. Average path length is relevant in many fields regarding real-life networks, including the design of routes in architectural design, signal integrity in communication networks, the propagation of diseases or beliefs in social networks or of technology in industrial networks. Moreover, many processes such as routing, searching, and spreading become more efficient when average path length is smaller. So far, much attention has been paid to the question of average path length. But the calculation of the characteristic path length in random networks is based on the renormalization group method or the mean field method, and there are no accurate and analytical expressions of average path length. For some deterministic models, there are some accurate solutions using various methods. Next, we find the exact analytical expression for the average distance $\mu ({G}_{m,t})$ of generalized Farey graphs ${G}_{m,t}$.

The definition of average path length in ${G}_{m,t}$ is as follows:

19 $$\mu ({G}_{m,t})=\frac{2{D}_{m,t}}{{n}_{m,t}({n}_{m,t}-1)},$$

where ${D}_{m,t}$ is total distance between all couples of nodes, i.e.

20 $${D}_{m,t}=\sum _{i\in {G}_{m,t},j\in {G}_{m,t},i\ne j}{d}_{i,j},$$

where ${d}_{i,j}$ is the shortest distance between node *i* and *j* in ${G}_{m,t}$.

The total distance ${D}_{m,t}$ in ${G}_{m,t}$ can be derived based on recursive characteristics of the network structure. The network has the following structure: ${G}_{m,t}$ is the merge of two sub-networks ${G}_{m,t}^{1/3,3}$ and ${G}_{m,t}^{2/3}$, where ${G}_{m,t}^{2/3}$ is the combination of two sub-networks ${G}_{m,t}^{1/3,1}$ and ${G}_{m,t}^{1/3,2}$. The three sub-networks ${G}_{m,t}^{1/3,i}$ (*i* = 1, 2, 3) are the same, so that we only need compute the total distance ${D}_{m,t}^{1/3}$ for any of the three sub-networks. The sub-networks have the same recursively increased structure. Moreover, just as for regular Farey graphs, all the nodes in these three sub-networks can be divided into three parts based on their distances to two hub nodes. With the help of the initial conditions ${D}_{m,t}^{1/3}$, we first deduce the total distance ${D}_{m,1}^{1/3}$ in a sub-network. Then we derived the total distance ${D}_{m,t}$ in ${G}_{m,t}$ from the merger of three sub-networks. The detailed derivation process is shown in the Supplementaly information.

From the last equation (A19) in the Supplementaly information, we deduce that:

21 $$\begin{array}{c}{D}_{m,t}=\frac{1}{3{(m+1)}^{2}{(2m-1)}^{3}}\{[(36{m}^{5}+54{m}^{4}-18{m}^{2})t+72{m}^{5}+81{m}^{4}-54{m}^{3}-63{m}^{2}]\\ \,\,\,\,{(4{m}^{2})}^{t}+(54{m}^{5}+9{m}^{4}-90{m}^{3}+9{m}^{2}+54m){(2m)}^{t}+18{m}^{5}-18{m}^{4}+36{m}^{3}+\\ \,\,\,\,36{m}^{2}+18m-18\}.\end{array}$$

By substituting it into equation (19), the averaged path length of generalized Farey graphs ${G}_{m,t}$ is calculated as follows:

22 $$\begin{array}{c}\mu ({G}_{m,t})=\frac{2}{3(2m-1){(m+1)}^{2}[9{m}^{2}{(2m)}^{2t}+(12{m}^{2}-15m){(2m)}^{t}+3{m}^{2}-9m+6]}\\ \,\,\,\,\{[(36{m}^{5}+54{m}^{4}-18{m}^{2})t+72{m}^{5}+81{m}^{4}-54{m}^{3}-63{m}^{2}]{(4{m}^{2})}^{t}+(54{m}^{5}\\ \,\,\,\,+\,9{m}^{4}-90{m}^{3}+9{m}^{2}+54m){(2m)}^{t}+18{m}^{5}-18{m}^{4}+36{m}^{3}+36{m}^{2}+18m-18\}.\end{array}$$

Given an infinite network size limit ($t\to \infty$), this becomes:

23 $$\mu ({G}_{m,t})\approx \frac{8{m}^{3}+12{m}^{2}-4}{6{m}^{3}+9{m}^{2}-3}t\approx \,\mathrm{ln}\,{n}_{m,t}.$$

Based on the above discussions, our model is a deterministic small-world network, because it is a sparse with exponential degree distribution, high clustering, short diameter and short average path length, which satisfy the four main necessary properties for small-world and scale-free networks.

## Conclusion

The scale-free characteristic emerges from the generalized Farey graphs, and this property is not found in the Farey graphs, because generalized Farey graphs have similar but not identical evolutionary mechanisms in comparison with Farey graphs. The degree distribution in genialized Farey graphs follows a power-law form where the exponent belongs to the interval [2, 3], while degree distribution is exponential in Farey graphs. The difference of topological properties among them are rooted in their different growth mechanisms. In other words, scale-free originates from the exponential increase mechanism of nodes’ degrees in generalized Farey graphs, while exponential degree distribution is caused by the linear incremental in Farey graphs.

The clustering coefficient tends to $1-\frac{3m+2}{2{m}^{2}-1}$ for large value of *t* in genialized Farey graphs. Therefore, for large value of *t* and *m*, the clustering coefficient approaches a constant value 1. That value in Farey graph is $\mathrm{ln}\,2$. Nodes’ degrees increase exponentially in generalized Farey graphs, while that growth in Farey graphs is linear, which leads to generalized Farey graphs having higher clustering coefficient than Farey graphs. The basic motif in each growth step is a triangle, which causes high clustering coefficients both in generalized Farey graphs and Farey graphs.

Average degrees are all small and approximately equal to 4 in generalized Farey graphs and Farey graphs. The proposed new models are sparse network as well. This is because the largest proportion of nodes in the two types of graphs only have a degree of 2.

We here also provided an appropriate example for a wide family of generalized Farey graphs, including the networks created by edge iterations^20^, or evolving networks with geographical attachment preference^21^, or the general geometric growth model for pseudo-fractal scale-free webs with parameters $q=2$ and $m=1$^22^. It should be mentioned that the final expressions in this paper are the extension of networks in ref^20–22^, so the explicit calculation presented here is a solution with more general usefulness.

The family of generalized Farey networks, being scale-free, with high clustering coefficients, small diameter and average path length, and small-world properties, successfully reproduces some remarkable characteristics in many natural and man-made networks and has special advantages in the research of some physical mechanisms such as random walks in complex networks. With the help of our results, the deterministic generalized Farey models will have unique virtues in contrast with more usually probabilistic approaches in understanding the underlying mechanisms between dynamical processes (random walks, consensus, stabilization, synchronization, etc.) applied to the structure of complex networks.

## Electronic supplementary material

Supplementary Information
