# Supplementary material for: Structure Properties of Generalized Farey graphs based on Dynamical Systems for Networks
Source: Sci Rep. 2018 Aug 15;8:12194. doi: 10.1038/s41598-018-30712-2 (PMC6093942; doi:10.1038/s41598-018-30712-2)
Supplement: Supplementary file 1 — Supplementary Information [file 41598_2018_30712_MOESM1_ESM.pdf]

Structure Properties of Generalized Farey graphs based on Dynamical Systems for Networks

Wenchao Jiang[1,*], Yinhu Zhai[2], Paul Martin[3], Zhiming Zhao[3]

[1] School of Computer, Guangdong University of Technology, Guangzhou, 510006, China

[*] Correspondence to: jiangwenchao@gdut.edu.cn

[2] School of Information Engineering, Guangdong University of Technology, Guangzhou, 510006, China

zhaiyh@gdut.edu.cn

[3] System and Network Engineering research group, Informatics Institute, University of Amsterdam, Science Park 904, 1098XH, Amsterdam, the Netherlands

p.w.martin@uva.nl z.zhao@uva.nl

Appendix

The detailed derivation of the average path length

1. We compute the sum of shortest paths in sub-network $G_{m,t}^{1/3,i}$, namely $D_{m,t}^{1/3}$.

As shown in Figure 3a in the paper, the two initial nodes in $G_{m,t}^{1/3,i}$ are X_i and Y_i, and $G_{m,t}^{1/3,i}$ is divided into three parts: $G_{m,t}^{1/3,i,x}$, $G_{m,t}^{1/3,i,y}$ and $G_{m,t}^{1/3,i,xy}$, on the basis of the distances from themselves to X_i or Y_i. Assuming the total number of nodes in sub-network $G_{m,t}^{1/3,i}$ is $n_{m,t}^{1/3}$, the numbers of nodes in $G_{m,t}^{1/3,i,x}$, $G_{m,t}^{1/3,i,y}$ and $G_{m,t}^{1/3,i,xy}$ are $n_{m,t}^{1/3,x}$, $n_{m,t}^{1/3,y}$ and $n_{m,t}^{1/3,xy}$, respectively. Like for a Farey graph, by the symmetry of generalized Farey graphs, one can infer:

$$n_{m,t}^{1/3,x} = n_{m,t}^{1/3,y}, \tag{A1}$$

$$n_{m,t}^{1/3} = n_{m,t}^{1/3,x} + n_{m,t}^{1/3,y} + n_{m,t}^{1/3,xy}. \tag{A2}$$

It then follows the three coupled relationships:

$$n_{m,t}^{1/3} = 2m \cdot n_{m,t-1}^{1/3} - 2m + 1, \tag{A3}$$

$$n_{m,t}^{1/3,x} = m \cdot n_{m,t-1}^{1/3,x} + m \cdot n_{m,t-1}^{1/3,xy} - m + 1 \tag{A4}$$

$$n_{m,t}^{1/3,xy} = 2m \cdot n_{m,t-1}^{1/3,xy} - m. \tag{A5}$$

By the help of the initial conditions $n_{m,1}^{1/3} = m+2$, $n_{m,1}^{1/3,x} = 1$ and $n_{m,1}^{1/3,xy} = m$, the solutions of equations (A3) to (A5) are then derived

$$n_{m,t}^{1/3} = \frac{m(2m)^t + 3m - 2}{2m-1}, \tag{A6}$$

$$n_{m,t}^{1/3,x} = \frac{(m^2+m)(2m)^t + (2m^2-m)(-m)^t + 3m^2 + 3m - 3}{3(m+1)(2m-1)} \tag{A7}$$

$$n_{m,t}^{1/3,xy} = \frac{(m^2+m)(2m)^t + (-4m^2+2m)(-m)^t + 3m^2 - 3m}{3(m+1)(2m-1)}. \tag{A8}$$

If we denote $\bar{G}_{m,t}^{1/3,i}$ exactly as $G_{m,t}^{1/3,i}$ but with the initial node X_i subtracted from itself,

and assume that $\overline{D}^{1/3}_{m,t}$ is the total of shortest paths from all the vertices of $\overline{G}^{1/3,i}_{m,t}$ to X_i, then we deduce the relationships, based on the detailed construction method in Figure 3a:

$$\overline{D}^{1/3}_{m,t} = 2m\overline{D}^{1/3}_{m,t-1} + 2m(n^{1/3}_{m,t-1} - 1) - 2m(n^{1/3,x}_{m,t-1})^2 - 2(m-1) \tag{A9}$$

and

$$\begin{aligned} D^{1/3}_{m,t} = {} & 2m\overline{D}^{1/3}_{m,t} + 2m(n^{1/3}_{m,t-1} - 1) - 2m(n^{1/3,x}_{m,t-1})^2 + \frac{m(m-1)}{2}\{2(2n^{1/3}_{m,t-1} \\ & -3)[2\overline{D}^{1/3}_{m,t} + 2(n^{1/3}_{m,t-1} - 1) - 2(n^{1/3,x}_{m,t-1})^2] - 2(2n^{1/3,x}_{m,t-1} - 1)(n^{1/3,x}_{m,t-1} \\ & - n^{1/3,xy}_{m,t-1}) - 2(n^{1/3,x}_{m,t-1} - n^{1/3,xy}_{m,t-1})(2n^{1/3}_{m,t-1} - 1) - 2(m-1)]\}. \end{aligned} \tag{A10}$$

By the help of initial conditions $\overline{D}^{1/3}_{m,1} = 2m + 2$ and $D^{1/3}_{m,1} = 2m^2 + 2m + 2$, we derive solutions for the two equations above

$$\begin{aligned} \overline{D}^{1/3}_{m,t} = {} & \frac{2}{9(m+1)(2m-1)^2}\{[(6m^3 + 3m^2 - 3m)t + 14m^3 + 4m^2 - \\ & 10m](2m)^t + (4m^3 - 4m^2 + m)(-m)^t + 18m^3 - 4m^2 + 9\}, \end{aligned} \tag{A11}$$

$$\begin{aligned} D^{1/3}_{m,t} = {} & \frac{2}{9(m+1)^2(2m-1)^3}\{[(15m^5 + 18m^4 + 6m^2)t + 20m^5 + 15m^4 - 30m^3 - 25m^2] \\ & (2m)^{2t} + [(14m^4 + 18m^3 - 6m)t + 54m^5 + 49m^4 - 42m^3 - 15m^2 + 22m](2m)^t \\ & + (34m^4 - 48m + 24m^2 - 4m)(-m)^t + 54m^5 - 72m^3 + 54m - 18\}. \end{aligned} \tag{A12}$$

The two-thirds of $G_{m,t}$, or the graph $G^{2/3}_{m,t}$, is the composite of $G^{1/3,1}_{m,t}$ and $G^{1/3,2}_{m,t}$, shown in Figure A1 as below. It is apparent that an initial node X_1 in $G^{1/3,1}_{m,t}$ and another initial node Y_2 in $G^{1/3,2}_{m,t}$ are combined into a vertex *Z*, while the other two initial nodes, Y_1 and X_2, in $G^{1/3,1}_{m,t}$ and $G^{1/3,2}_{m,t}$ are linked directly.

2. The relationship of the sum of shortest paths $D^{2/3}_{m,t}$ in $G^{2/3}_{m,t}$ is derived as below.

$\bar{D}^{1/3,i}_{m,t}$ is the total of all shortest paths between an initial node and the other nodes in $G^{1/3,i}_{m,t}$, in which $\bar{D}^{1/3,1}_{m,t} = \bar{D}^{1/3,2}_{m,t} = \bar{D}^{1/3}_{m,t}$. Similarly, we denote $\bar{D}^{2/3}_{m,t}$ as the sum of shortest paths between an initial node and the other nodes in $G^{2/3}_{m,t}$. Obviously, in Figure A1, if the total shortest paths from all nodes in $G^{1/3,2}_{m,t}$ to an initial node *X* go through vertex *Z*, then

$\bar{D}^{2/3}_{m,t} = \bar{D}^{1/3,1}_{m,t} + \bar{D}^{1/3,2}_{m,t} + 2n^{1/3}_{m,t} - 2$. However, the shortest paths from the vertices in $G^{1/3,2,y}_{m,t}$ to Z should pass through vertex Y, but not through Z, so that the number of surplus or additional paths is $2n^{x}_{m,t}$, and hence we get:

$$\bar{D}^{2/3}_{m,t} = \bar{D}^{1/3,1}_{m,t} + \bar{D}^{1/3,2}_{m,t} + 2n^{1/3}_{m,t} - 2 - 2n^{1/3,x}_{m,t}. \tag{A13}$$

The sum of shortest paths in $G^{2/3}_{m,t}$ includes three parts: $D^{1/3}_{m,t}$ in $G^{1/3,1}_{m,t}$, $D^{1/3}_{m,t}$ in $G^{1/3,2}_{m,t}$ and the paths Ω_{tk} between all nodes in $G^{1/3,1}_{m,t}$ and $G^{1/3,2}_{m,t}$. If all the shortest paths pass through vertex Y, Ω_{tk} consists of $2\,(n^{1/3}_{m,t} - 1)\overline{D}^{1/3}_{m,t}$. But, because nodes X and Y are neighbors, the shortest paths between nodes in $G^{1/3,1,y}_{m,t}$ and $G^{1/3,2,x}_{m,t}$ should go by X and Y, but not by Z, so that the number of supernumerary paths is $2(n^{1/3,x}_{m,t})^2$. We then obtain the sum of shortest paths $D^{2/3}_{m,t}$

$$D^{2/3}_{m,t} = 2D^{1/3}_{m,t} + 2(n^{1/3}_{m,t} - 1)\overline{D}^{1/3}_{m,t} - 2(n^{1/3,x}_{m,t})^2. \tag{A14}$$

For the initial conditions $\overline{D}^{2/3}_{m,1} = 6m + 4$ and $D^{2/3}_{m,1} = 18m^2 + 12m + 6$, the solutions of equation (A13) and (A14) are calculated

$$\begin{aligned}\overline{D}^{2/3}_{m,t} = {} & \frac{2}{9(m+1)(2m-1)^2}\{[(12m^3 + 6m^2 - 6m)t + 40m^3 + 14m^2 - 26m] \\ & (2m)^t + (-4m^3 + 4m^2 - m)(-m)^t + 36m^3 - 18m^2 - 27m + 18\}\end{aligned} \tag{A15}$$

$$\begin{aligned}D^{2/3}_{m,t} = {} & \frac{2}{9(m+1)^2(2m-1)^3}\{[(24m^5 + 36m^4 - 12m^2)t + 46m^5 + 48m^4 - 42m^3 - 44m^2] \\ & (2m)^{2t} + (8m^5 - 12m^4 + 6m^3 - m^2)m^{2t} + [(12m^5 + 18m^4 + 6m^2)t + 106m^5 + \\ & 75m^4 - 144m^3 + 29m^2 + 54m](2m)^t + (-8m^5 + 6m^3 - 2m^2)(-2m^2)^t + (-16m^5 \\ & + 24m^4 - 8m^3 + 2m^2)(-m)^t + 72m^5 - 27m^4 - 108m^3 + 45m^2 + 54m - 27\}.\end{aligned} \tag{A16}$$

How to make $G^{1/3,3}_{m,t}$ and $G^{2/3}_{m,t}$ merge into $G_{m,t}$ is an important concern. It is apparent that $G_{m,t}$ is created by merging the two edges, between the two initial nodes in $G^{1/3,3}_{m,t}$ and $G^{2/3}_{m,t}$, into one edge from X to Y. We demonstrate the two main processes of it in Figure A2 as below. The first step, shown in Figure A2a, is the merging of nodes X and X_3 into X, the second step, in Figure A2b, is the merging of Y and Y_3 into Y.

3. We deduce the total shortest paths $D_{m,t}$ in $G_{m,t}$.

Suppose that $\overline{D}_{m,t}$ is the total shortest paths between all nodes excluding an initial vertex X in $\bar{G}_{m,t}$ and X. From Figure A2a, $\overline{D}_{m,t}$ is exactly the sum of $\overline{D}^{1/3}_{m,t}$ and $\overline{D}^{2/3}_{m,t}$. But, we can find that the shortest path between X to Y are calculated twice in Figure A2b, so that:

$$\overline{D}_{m,t} = \overline{D}^{1/3}_{m,t} + \overline{D}^{2/3}_{m,t} - 2 \cdot \tag{A17}$$

Based then on Figure A2a, the sum of the shortest paths in the united graphs is $D_{m,t} = D^{2/3}_{m,t} + D^{1/3}_{m,t} + (n^{2/3}_{m,t} - 1)\overline{D}^{1/3}_{m,t} + (n^{1/3}_{m,t} - 1)\overline{D}^{2/3}_{m,t}$. However, after the merging of nodes Y and Y_3 into Y in Figure A2b, all the shortest paths between nodes in $\bar{G}_{m,t}$ and node Y will disappear. The number of obsolescent shortest paths then is $\overline{D}^{1/3}_{m,t} + \overline{D}^{2/3}_{m,t} - 2(n_{m,t} - 1)$ and every shortest path between $G^{2/3,xy}_{m,t}$ and $G^{1/3,3,y}_{m,t}$, $G^{1/3,3,xy}_{m,t}$ and $\bar{G}^{2/3,y}_{m,t}$, and $G^{1/3,3,y}_{m,t}$ and $\bar{G}^{2/3,y}_{m,t}$ will shorten by 2, 2 and 4, respectively. The surplus paths are therefore $2n^{2/3,xy}_{m,t}(n^{1/3,x}_{m,t} - 1) +$ $2n^{1/3,xy}_{m,t}n^{2/3,x}_{m,t} + 4n^{2/3,x}_{m,t}(n^{1/3,x}_{m,t} - 1)$, noting that $n^{1/3,xy}_{m,t} + 2n^{1/3,x}_{m,t} = n^{1/3}_{m,t}$ and $n^{2/3,xy}_{m,t} + 2n^{2/3,x}_{m,t} = n^{2/3}_{m,t}$. As such, the sum of shortest paths of $G_{m,t}$ satisfies the recursive relationship below:

$$\begin{aligned} D_{m,t} &= D^{2/3}_{m,t} + D^{1/3}_{m,t} + (n^{2/3}_{m,t} - 2)\overline{D}^{1/3}_{m,t} + (n^{1/3}_{m,t} - 2)\overline{D}^{2/3}_{m,t} \\ &+ 2 - 2n^{2/3,xy}_{m,t}n^{1/3,x}_{m,t} - n^{2/3,x}_{m,t}n^{1/3}_{m,t}. \end{aligned} \tag{A18}$$

By the help of initial condition $D_{m,1} = 18m^2 + 18m + 6$, we deduce that:

$$\begin{aligned} D_{m,t} = &\frac{1}{3(m+1)^2(2m-1)^3}\{[(36m^5 + 54m^4 - 18m^2)t + 72m^5 + 81m^4 - 54m^3 - 63m^2] \\ &(4m^2)^t + (54m^5 + 9m^4 - 90m^3 + 9m^2 + 54m)(2m)^t + 18m^5 - 18m^4 + 36m^3 + \\ &36m^2 + 18m - 18\}. \end{aligned} \tag{A19}$$

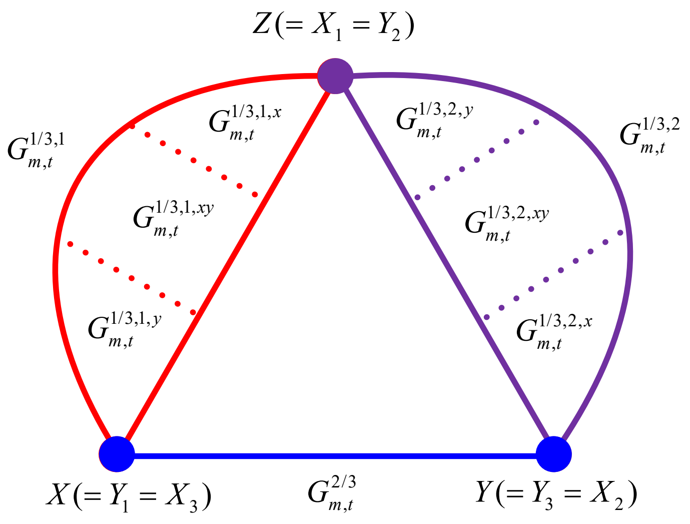

Figure A1. The construction schematic diagram of $G_{m,t}^{2/3}$ by composing $G_{m,t}^{1/3,1}$ and $G_{m,t}^{1/3,2}$

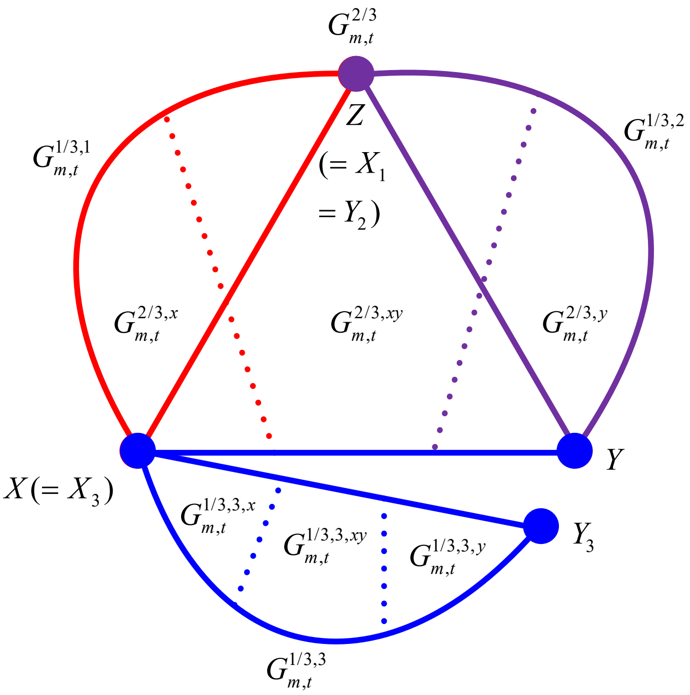

a. The merging of nodes X and X_3.

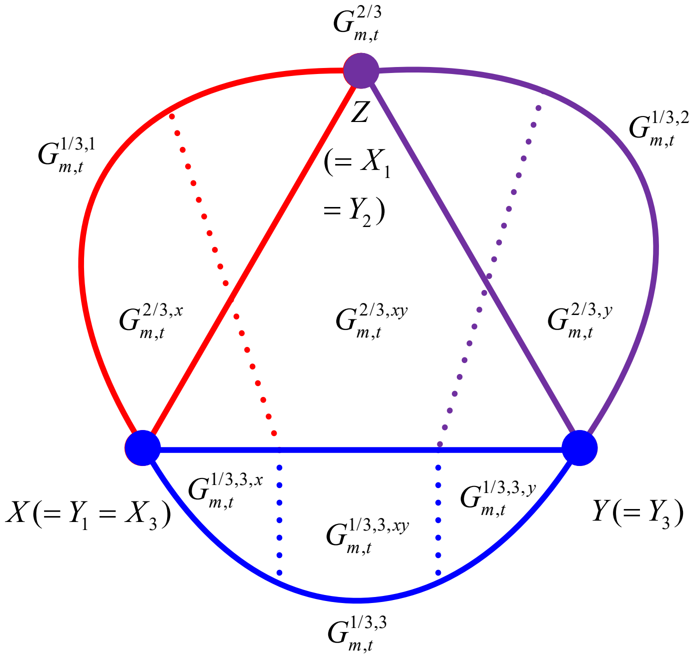

b. The merging of nodes Y and Y_3.

Figure A2. The schematic diagram of the two construction processes of $G_{m,t}$ by composing $G_{m,t}^{1/3,3}$ and $G_{m,t}^{2/3}$.
